# Busting myths in online education: Faculty examples from the field

**DOI:** 10.1017/cts.2021.808

**Published:** 2021-06-28

**Authors:** Katherine Guevara, Layla Fattah, Anamara Ritt-Olson, Pai-Ling Yin, Lesley Litman, Samira S. Farouk, Rebecca O’Rourke, Richard E. Mayer

**Affiliations:** 1 Southern California Clinical and Translational Science Institute (SC CTSI), University of Southern California, Los Angeles, CA, USA; 2 ConduITS, The Institutes for Translational Sciences, Icahn School of Medicine at Mount Sinai, New York, NY, USA; 3 Department of Preventive Medicine, Keck School of Medicine, University of Southern California, Los Angeles, CA, USA; 4 Lloyd Greif Center for Entrepreneurial Studies, Marshall School of Business, University of Southern California, Los Angeles, CA, USA; 5 School of Education, Hebrew Union College-Jewish Institute of Religion, New York, NY, USA; 6 Division of Nephrology, Department of Medicine, Icahn School of Medicine at Mount Sinai, New York, NY, USA; 7 Institute of Medical Education (LIME), School of Medicine, University of Leeds, Leeds, UK; 8 Psychological and Brain Sciences, University of California, Santa Barbara, CA, USA

**Keywords:** Online education, case study, appreciative inquiry, Universal Design for Learning, UDL, trauma-informed teaching, Scholarship of Teaching and Learning, SoTL, Self-Determination Theory, SDT, empathy

## Abstract

The shift in learning environments due to the COVID-19 pandemic necessitates a closer look at course design, faculty approaches to teaching, and student interaction, all of which may predict learner achievement and satisfaction. Transitioning to an online environment requires the reinvention, reimagining, and applying of “e-flavors” of general learning theory. With this shift to online learning comes the opportunity for misunderstandings and “myths” to occur, which may stand in the way of faculty embracing online learning and fully realizing its potential. This article seeks to address several myths and misconceptions that have arisen in higher education during the rapid shift to online teaching and learning. While not comprehensive, these myths represent a snapshot of common challenges. These are we can transfer our in-person course design to online; adult learners do not need an empathetic approach; and online teaching and learning is socially isolating. Through an appreciative inquiry framework, we present each myth in the context of relevant literature and invite faculty with varied online teaching experience to share their own case studies that illustrate how they have “busted” these myths with the goal to identify existing examples of locally effective practices for the express purpose of replication that leads to positive change.

## Introduction

Across higher education, the COVID-19 pandemic has necessitated a rapid shift from face-to-face to online learning. For some programs and schools with an existing online presence, this transition came as a subtle shift in emphasis, while for others, it created a seismic shift in both culture and technology [1]. This shift in learning environments necessitates a closer look at course design, faculty approaches to teaching, and student interaction, all of which may predict learner achievement and satisfaction [2].

Understandably, faculty new to online teaching and learning were, and many still are, unsure of the approaches to take and find themselves learners anew, often trying to tackle a steep learning curve in real time [3]. Making this transition requires learning theory knowledge and applying it through technology in an accessible and inclusive way. For many, this highlighted that some in-person teaching practices could not be easily or effectively shifted to an online environment.

It is important to note that online learning is not a single entity as it offers an ever-expanding collection of pedagogies, constructs, modalities, and technologies. There is no single approach to providing effective and engaging online education [4]. Education research has evidenced no pedagogical models that are entirely exclusive to an online environment [5] which means best practices for designing and delivering effective teaching are essentially the same across modalities [2]. Instead, an online environment requires the reinvention, reimagining, and applying of “e-flavors” of general learning theory [5]. It requires new ways of thinking about teaching and learning [6]. The media or platform itself does not foster learning, but instead it is the underpinning instructional method or pedagogy that promotes online learning [7].

With this shift to online learning comes the opportunity for misunderstandings and “myths” to occur, which may stand in the way of faculty embracing online learning and fully realizing its potential. Inspired by Clark Quinn’s Millennials, Goldfish & Other Training Misconceptions: Debunking Learning Myths and Superstitions [8], this article seeks to address several myths and misconceptions that have arisen in higher education during the rapid shift to online teaching and learning as a result of the COVID-19 pandemic. These are

- Myth 1-We can transfer our in-person course design to online;
- Myth 2-Adult learners do not need an empathetic approach;
- Myth 3-Online teaching and learning is socially isolating.

We present each myth in the context of relevant literature and invite faculty with varied online teaching experience to share their own case studies that illustrate how they have “busted” these myths, in many cases experiencing an “ah-ha” moment or a shift in perspective themselves in relation to online teaching and learning, many times a result of their own learning about pedagogy. The authors recognize the myths presented in this article are not comprehensive yet represent a snapshot of common challenges faculty have faced in an online education setting. The myths we discuss cover course design, faculty approach to teaching, and fostering interaction and community online. These are some of the key tenants of online learning, and the case studies faculty provide illustrate how they have overcome these myths to develop and deliver evidence-based online education that has engaged, challenged, and supported their students across a range of institutions and disciplines.

This approach follows an appreciative inquiry framework whose hallmark goal is the identification of existing examples of locally effective practices for the express purpose of replication that leads to positive change. Notably, appreciative inquiry was born in the 1980s from the field of organizational behavior and rooted in positive psychology as a strength-based rather than deficit-focused approach intentionally highlighting what works in order to do more of it with the aim of effecting positive change [9–11]. In keeping with the theme of busting myths, using such an affirmative approach instead assumes the positive, emphasizes strengths, and places value on collective understanding of what defines or makes up the best in online teaching and how to work toward dispelling myths that do not. To that end, we provide case study examples in the application of research-based teaching practices faculty have found to have a positive effect on online teaching and learning; reporting such learning is essential to achieve sustained change in higher education [12].

These case studies collected through appreciative inquiry evidence the nonexhaustive breadth of approaches to online learning. This myth-busting inquiring is about “generating and inspiring new ideas, visions, and stories that can potentially lead to action” [13]. We hope that the real-world examples presented will inspire other colleagues to share their effective practices and relate to a community of colleagues united in their mission to provide quality online education by following best practices in online course design and teaching.

## Myth 1: We Can Transfer Our in-Person Course Design to Online

A common misconception for faculty approaching online teaching for the first time is trying to emulate traditional face-to-face education strategies in an online environment. It is essential to highlight that designing and teaching courses are a research-based science of their own and that designing for in-person instruction differs from designing online in many regards, though the basis in sound pedagogy is universal despite the modality. The media or platform does not in itself foster learning, but instead it is the underpinning instructional method or pedagogy that determines whether students learn [7]. At the outset, the approach should be to consider how technology is best employed to support learning, rather than starting with the technology and working backward.

While faculty are undeniably renowned experts in their disciplines, many lack formal education in how adults learn, or professional development regarding how to design and teach a course using evidence-based methods [3, 14]. Many institutions recognize the need to invest in advancing the Scholarship of Teaching and Learning (SoTL) [15] to support the design and teaching of courses using evidence-based methods, including for the already mature field of online education. This is often accomplished with the support of instructional designers and other education experts [16]. An unanticipated benefit of the shift to online education has been an increase in faculty interest, training, and innovation in teaching generally, and online course design and instruction specifically. As educators continue to innovate in an online space, we see the growing scope and potential for online education to change the way we teach and learn.

The following two case studies present examples of applying science in an online context and describe faculty experiences on this journey.

### Case Study: Content Creation for Effective Learning

#### Richard Mayer, Distinguished Professor of Psychology at the University of California, Santa Barbara

Educational psychologist Richard Mayer is a leading expert in cognition and learning theories, particularly in the design of educational multimedia. He seeks to base his instructional practice on evidence-based principles grounded in a cognitive theory of how people learn [17–19]. Mayer has known the value of the science of learning to contribute to the design of learning environments – including online ones – for years: “This is not a new idea in education and dates back more than 100 years to calls by E. L. Thorndike and William James to base education on the science of how the human mind works.”

Mayer’s Principles of Multimedia underpin content creation for successful instruction, whether that be designing presentation slides, creating recorded videos or structuring live session work. These materials can be used synchronously, for example, when an instructor is presenting information live during class time, or asynchronously, for example, for recorded sessions that the students can view in their own time. This case study illustrates a number of Mayer’s principles and how these can be employed to guide the creation and delivery of course content.

The principles (several presented in Table 1 with examples of aligned applications) are based on both Mayer’s research [18,19] and cognitive load theory [20], which states the importance of reducing the amount of mental effort being used by the learner in their working memory or stored in short-term memory. In an online environment, it can be more challenging than usual for learners to focus on the material being presented in part due to the context clues the brain is lacking that typically help it process information when face-to-face; this is sometimes referred to as “Zoom fatigue” and has a neuropsychological explanation [21]. For this reason, creating content that supports cognitive processing, or the ability of a learner to obtain, store, and retrieve information, is arguable more critical online.

**Table 1.** Five of Mayer’s principles of Multimedia *[18]*

Principle	Description	Theory/Process	Examples of Application
For reducing extraneous processing that does not serve the instructional goal			
Coherence	Students learn better when instructors exclude extraneous words, pictures, and sounds	Cognitive Load Theory	Less is more; create minimal slide decks with fewer slides and less text on each slide; only incorporate images/audio when they support the teaching of a concept
Signaling	Students learn better when instructors use cues that highlight the organization of essential material	Cognitive Load Theory	Use an agenda or outline; call attention to key terms such as with highlighting or bolded text
For managing essential processing needed to acquire or complete the essential information			
Segmenting	Students learn better when instructors present a multimedia lesson in user-paced segments rather than as a continuous unit	Microlearning Flipped Classroom Intrinsic Motivation	Break up one longer lecture into several shorter ones
For promoting generative processing to make sense of the material and store it (long-term memory) more easily			
Embodiment	Students learn better when instructors present a multimedia lesson from a first-person perspective that uses human gestures, expression, and body movement	Generative Cognitive Processing	Be yourself; frame recordings to capture body language
Generative Activity	Students learn better when instructors use active learning that allows for application, practice, and construction	Active Learning Constructivism	Pause traditional lecture often to do planned interactive activities

In an online environment particularly, it can be challenging for learners to deal with extraneous cognitive processing, which is the unproductive mental effort created by delivering course content with extraneous text and images. Mayer recommends limiting extraneous cognitive processing by designing presentation material that eliminates extraneous and redundant sounds, words, or images and highlights important terms and images to help reduce distraction for the learner. Faculty have reduced both the number of slides and the amount of content on each slide keeping only essential text and only those visuals that genuinely add to learning rather than distracting from or not relating to it. They also provide visual cues, for example, by highlighting or bolding sections, to indicate what of the presented information should be the focus of most attention.

Alongside this, a conscious effort should also be made to manage essential processing, which is the effort required to learn the core material. This can be achieved by breaking the lesson into smaller segments rather than one, long, continuous unit. This approach can be particular valuable when taking a flipped classroom approach, whereby students consume lecture material in their own time and work on more dynamic, problem-solving activities during synchronous teaching time [22]. The asynchronous online learning can be presented as short segments that have been “chunked” into smaller, more focused recorded videos that can be paused and re-watched at the learner’s own pace. This is sometimes referred to as “micro-learning” which improves both the retention and transfer of learning [23]. This has the added benefit that learners can control the pace much easier in a recorded lecture than a live one which can help them get through the material or complete a task.

Finally, a core aim of any approach to learning is for learners to make sense of material and store it in their long-term memory. Generative cognitive processing is the mental effort it takes to make sense of the task and store it in long-term memory. During live sessions or synchronous work, generative cognitive processing can be fostered in part through increasing the amount of active learning. To improve video effectiveness, Mayer counsels onscreen instructors should work hard to display human-like gestures, facial expression, and body movement during lecturing and to shoot instructional videos from a first-person perspective rather than a third-person perspective. Active learning focus, based in constructivism [24], focuses on breaking up traditional lecture time by having students apply and practice what they are learning, often collaboratively, by engaging in in-class work activities. This requires inserting prompts for practical learning activities such as generating a summary, explanation, or drawing during learning. For example, during Zoom group breakout sessions, provide clear instructions or prompts and job roles for each member to accomplish. Students are held accountable for producing a work product or deliverable that is often submitted or presented by the end of class time.

The overarching message is that the way educational material is presented to learners significantly impacts their retention and takes careful planning. Mayer recommends “carefully choosing principles that appear to be most useful, pilot testing them with learners, and adjusting them until you are comfortable with them.”

### Case Study: Faculty as Students of Online Pedagogy

#### Lesley Litman, Director of the Executive M.A. Program in Jewish Education and Online Instructional Support Coordinator at Hebrew Union College-Jewish Institute of Religion

Lesley Litman and the Online Learning Task Force of HUC-JIR realized their faculty colleagues needed programmatic, system-wide professional development to quickly learn and put into practice some of the science behind online course design and teaching, including Mayer’s Principles, and learn the digital tools required for facilitating it. “Bringing an entire faculty online in a short period seemed like a daunting task. Initially, we required the faculty to participate in a two-week online course on Online Teaching and Learning. About 60% of faculty participated in some way. The goal was to help them acquire basic pedagogic tools (articulating objectives, aligning assessment with objectives, assessment design) as well as tools unique to online teaching (designing a synchronous Zoom session, creating effective videos).” While Litman’s faculty were subject-matter experts with years of in-person teaching experience, most had never studied the SoTL. The majority of her faculty were brand new to teaching online and were also switching their Learning Management System (LMS).

Learning both a new pedagogy and a new LMS, coupled with the added pressure of needing to learn them quickly, proved overwhelming. Through reflection, feedback, and conversations, Litman realized HUC-JIR needed a conceptual framework to support faculty at this particular moment of their vulnerability as learners when experts find themselves novices again and under pressure to make quick progress at something new and scary during times that were also unprecedented and fearsome. She decided on a reflection-based approach: “At the core of our practice is reflection. Using design thinking tools, the teaching and learning practices we used with our faculty were iterative, based on pilot actions, feedback, reflection, and ideation, leading to new responses and implementation. A second overarching component of our practice, also at the core of ‘design thinking’ is the notion of human-centered design. These conceptual frameworks (reflection and human-centered design) underpin the vast majority of our decisions and actions.” Litman’s conceptual framework for supporting faculty who are learning about teaching online may be applicable to others creating such programming for faculty who are learning about other aspects of teaching including when in-person learning returns.

Given Litman’s institution’s size, with about 130 faculty, a more individualized approach was possible. She provided multiple and varied faculty opportunities to learn, including online courses, one-on-one sessions, drop-in hours, regular email communication with tips and resources, and short, frequent workshops. The faculty were responsive and grateful for the support, but was it working? Did faculty learn about the science of online teaching and did learning translate to improved course design, teaching, and student success? Litman asked the students, whose feedback consistently pointed to challenges in understanding online course expectations and navigating the course. Continued faculty course design and teaching support were needed to address these student concerns and improve their learning experience. Litman started by having faculty individually meet with her to go through their online course in the LMS, which they deemed a “site check,” and formulate approaches to improve practice. “Most importantly, the [site checks] included conversations about what faculty were trying to achieve in their teaching and for their learners, where their concerns lay, their technological skill level, and their capacity to acquire the skills needed for success. These conversations have become a key tool in our faculty teaching and learning practice. The process of clarifying goals and objectives was powerful for faculty and enabled us to understand the tools and techniques they required to accomplish their aims more deeply. In response, we began to offer individual meetings and consultations, short (30-minute) sessions on particular aspects of online learning (e.g., The Brain on Zoom, Creating and Grading Online Assignments), adding Online Teaching and Learning Tips to bi-weekly emails while simultaneously creating an extensive resource area with simple, easily adopted techniques.”

Not surprisingly, Litman’s parting advice for working with faculty as learners of online pedagogy echoes the very same best practices we would use with any adult learners: “Our ‘students’ were teachers, both long-term (tenured and other) faculty and adjunct. As with any inclusive learning environment, we recommend providing multiple options for learning. Some learners like to experiment independently, refining their course sites over time, while others prefer to map everything out and be highly structured. The former may be fine with group courses and online instruction videos, and so forth. The latter may require more directed support or supplemental individualized tutoring. Our respect for how they learn, their strengths, and their fears are critical for their growth. We should not minimize the emotional component and anxiety resulting from learning, adapting, and succeeding in this new milieu.”

As Mayer and Litman’s case studies reveal, faculty could not just transfer what they did in-person and do it online because the design of effective online learning, like all learning, is evidence-based and principles and practices inform its careful planning, even during a pandemic or other emergency when that planning must take place at warp speed. Litman’s reference to the emotional component and resulting anxiety from learning not only in a new way but also while under pandemic circumstances holds true for all students whether those students happen to be faculty themselves or the learners who faculty teach and brings us to our next myth.

## Myth 2: An Empathetic Approach is Not Needed for Adult Learners

When we think of online learning, empathy may not be the first word to come to mind. When learning transitioned fully to online at the start of the pandemic, maintaining teaching schedules and academic rigor were highlighted as a high priority. It was quickly apparent that serious concern also needed to be given to student well-being and inclusivity, which was now as, if not more important, for remote learners struggling to adapt to an online learning space in addition to the other ongoing challenges of a pandemic [25, 26]. Taking a human-centered design approach puts empathy at the heart of the learning experience [27] and incorporates strategies from both Universal Design for Learning [28] and the trauma-informed teaching perspective [29].

Using scientific evidence into how humans learn, CAST, a nonprofit research and development organization, took those insights and created the Universal Design for Learning (UDL) framework and UDL guidelines to help make learning more inclusive [28]. Structuring teaching with the UDL framework provides the learner with multiple ways to engage by offering information in various formats and using diverse methods for the action and expression of learning. Doing so activates affective (the “why” of learning), recognition (the “what” of learning), and strategic networks (the “how” of learning) in the brain. Keeping learners’ diversity in mind, including how the brain learns, and their ability to access and process materials, makes for inclusive learning that proves more not only more effective but also more empathetic.

A further emerging approach, based on the construct of empathy, is trauma-informed teaching. This provides another way to underpin learning with empathy, while maintaining academic rigor [29]. Trauma-informed principles and practices that enhance the learning environment’s emotional safety include co-creating and implementing class policies to foster such an environment, doing regular check in with students, following up with students after class, collecting regular student feedback and integrating their suggestions, allowing students to tend to their emotional needs, discussing difficult material with time to process it and ability to opt out of discussing it, and acknowledging emotional overwhelm [30].

Following are two case studies with faculty attempting to create a more empathetic approach to online teaching, underpinned by the principles and practices of UDL and trauma-informed teaching.

### Case Study: Recognizing the Student Struggle and Checking in

#### Pai-Ling Yin, Associate Professor of Clinical Entrepreneurship and Director of the Technology Commercialization Initiative at the University of Southern California Marshall School of Business

Pai-Ling Yin recognizes that the emotional component and often traumatic anxiety resulting from learning, adapting, and succeeding in this new milieu cannot be minimized: “The rapid and total conversion of our teaching to online has been a shock to everyone’s system, compounded with the health and economic crisis around us. We still have a long way to go, and we cannot forget the health and sanity not only of our students but the faculty struggling to become familiar with this new environment as well.”

Taking an empathetic approach means addressing student challenges and emotions commonly tied to traumatic events openly right from the start and perhaps humanizing ourselves in a way we might not think to do in person. “I think you have to do a lot at the beginning to ask how the students are doing and feeling about the online environment and make sure there is a way they can communicate if they are having any difficulties accessing the class. I think they also appreciate the faculty acknowledging that online learning is very new for everyone and not always easy: we are all still learning.” Yin giving students time to process what is happening enhances the learning environment’s emotional safety.

Yin uses the flipped classroom model having students complete work before class in preparation for discussion and active learning activities during class time. She follows the UDL framework by providing materials in various formats and multiple outlets to express learning and the processing of the experience. “I know that during COVID, there is much fatigue from constantly being in a meeting and from even watching videos asynchronously, so I also include audio versions of the readings. However, the flipped classroom relies critically on students doing the work beforehand, so I make sure that they do a pre-class poll [related to their process of completing the assignment, pain points, and to establish how long it took them to complete the assignment] and I draw on their answers to that poll for our class discussion [and to make course updates and corrections]. I make a deliberate effort to make sure I get each person involved over the course in speaking during our online class.” At the start of synchronous sessions, Yin asks students a warm-up question as a self-assessment about how present they feel for the day’s class: ready to learn, unable to focus today, etc and noticing patterns in their responses can indicate personal outreach to a student may be needed.

Employing weekly pre-class polls via the online polling platform, Qualtrics, allows Yin to get regular feedback from students about how the class is going. She also uses pre-class worksheets to then facilitate in-class group work to help students share and compare answers while working together in small groups. “Breakout rooms are great! It is so hard to schedule breakout rooms normally because of the constraint on physical space, and I lose a lot of time asking students to transition from [physical] breakout rooms to the main classroom. It is also sometimes too loud for students to do work in a physical room in small groups. [Now], I can monitor progress through shared Google Docs.” Yin’s dedication to using a plethora of constant feedback loops helps her check in with student processing of not only the course content but also their emotions.

Yin’s academically rigorous course remains so online, but its facilitation now supports an empathetic approach to students though UDL. Our next case study shows examples of how checking in with ourselves – our intrinsic motivation and self-determination – forms part of a compassionate approach.

### Case Study: Toward a More Compassionate Approach

#### Anamara Ritt-Olson, Assistant Professor of Clinical Preventive Medicine, Health Education and Promotion Concentration at the University of Southern California Keck School of Medicine

Anamara Ritt-Olson uses self-determination theory to focus on intrinsic motivation – the need for learners to feel autonomy, mastery, and connectedness. “Self-Determination Theory (SDT) is a framework of smaller theoretical principles that explain and help learners achieve intrinsically motivated connection with the course content. SDT posits that people are driven by the need to be autonomous, competent, and feel a sense of relatedness [31]. A powerful course is designed to help students feel like they can work in a way that feels good to them (autonomous), that they can accomplish the course tasks (competent), and one that encourages personal connections (relatedness). This approach is not technology-dependent, but technology and distant learning practices can help achieve these goals more readily than in-person instruction.”

Ritt-Olson carries out self-determination theory in her teaching practice specifically by addressing the psychological and socio-emotional needs of the learner from a trauma-informed perspective, embedding learner choice tied to their prior knowledge, personal experiences, and interest from UDL, and using transparent practices of how their work will be assessed: “Fostering autonomy online is easily done by giving a choice to learners, a range of topics or types of projects that students can do. Fostering competence is done through clear expectations and well-worded materials. Utilizing assessment rubrics and fair grading practices also help and can easily be done online. Relatedness is trickier. It relies on good social skills and social intelligence. Most essentially is the relatedness aspect of the SDT. While building connection online may seem a daunting task, I enjoy the connection to students across [this type of] digital divide.”

Ritt-Olson has identified that the online environment has provided advantages to connecting with students in terms of becoming what she has coined a more “compassionate educator.” “Even exceptional teachers forget to connect across the divide with the people that have joined them in the classroom. They can be uncomfortable acknowledging people on a personal level, but we all need to feel seen. The online environment helps me track students’ interests across discussion boards, the Zoom classroom helps me keep track of names, and the chat window allows off-topic personal thoughts to be shared in a way that can be non-threatening. When the learner’s camera is on, it allows me to more easily view facial reactions and cues to better connect with students.” However, there is understanding too, for times and reasons when cameras cannot be on and learners are not able to share.

It has been a plus for Ritt-Olson to become more compassionate, which others might confuse with not maintaining academic rigor or incorrectly assume the impossibility of providing both; however, “Grounding in a place of seeking to satisfy the learner’s psychological needs in addition to educational attainments allows naturally for a compassionate pedagogy to be followed and a better learning environment. I think relatedness takes more practice and grace as you find authentic ways to acknowledge the learners in your class.” The concept of relatedness includes maintaining a teaching presence online [32]. This online presence means assuming different roles. “You create content, facilitate discourse and do direct instruction. You need to move fluidly through all three roles consistently throughout teaching the course. Asynchronous courses can be created to allow the student a more significant presence with the material: animations, lectures that ask for comments or feedback as you watch, all help. Discussion boards and student facilitation leaders allow for material processing, but instructors must monitor and add comments to those boards to keep them active. Synchronous learning is often more comfortable with that face to face, even through Zoom interaction.” All these different ways Ritt-Olson provides for students to be present with, process, and apply learning material not only give her a way to relate to students but also follow UDL principles.

Ritt-Olson’s compassionate pedagogy, in many ways, fosters connection even at a digital distance. These connections can include providing guidance on engagement with course content; opportunities for feedback; timely, specific, clear, and consistent feedback; accessibility and responsiveness during interactions with instructors and peers, and using technology to facilitate interaction [33–35]. Such empathetic and compassionate strategies as Yin’s and Ritt Olson’s lead us to our third and final myth.

## Myth 3: Online Teaching and Learning is Socially Isolating

Social isolation is perhaps the most difficult of our myths to bust, but we hope to illustrate how online learning creates socialization and connection opportunities that may not be possible otherwise. Learning through social connection is at the very heart of social constructivist theories of learning. It is widely acknowledged that learning is social in nature [36]. Through conversation, discussion, and debate, new learning is fostered among both students and between instructors and students [37].

Perhaps unsurprisingly, evidence suggests that online learning is more likely to make students feel disconnected than face-to-face learning [38]. However, some factors are strongly correlated with a sense of community. Facilitation of discussion is one factor most strongly associated with students’ sense of connection, indicating that online learning should consciously emphasize and foster approaches that encourage conversation with both faculty and peers [39]. Online learning requires adjustments by instructors as well as students for successful interactions to occur. However, fostering this connection is worthwhile, as students who fail to make online connections with other learners often report feelings of isolation and increased stress when compared to their counterparts [40]. Therefore, synchronous and asynchronous approaches should both be designed purposefully to engage learners in the sense of community.

The following case studies provide examples of how social connection can be fostered despite the challenges of an online environment.

### Case Study: Using Social Media to Expand Social Networks

#### Samira Farouk, Assistant Professor in Medicine/Nephrology and Medical Education, Icahn School of Medicine at Mount Sinai

Samira Farouk has been part of a recurring nephrology virtual journal club on Twitter, (#NephJC) which brings together participants from across the world who are interested in discussing relevant and timely nephrology topics [41, 42]. She explains, “the journal club brings together a wide range of people, from students and fellows to professors and presidents of national organizations. What started out as mainly nephrology attendings and some fellows grew to see more students and residents involved. We’ve worked to normalize it for them to feel more comfortable joining the discussion. Sometimes, it’s harder to speak up in real life, especially if you are shy or not very confident. In an online space, regardless of who you are, some may be freer to actively participate and contribute to the discussion with less fear of embarrassment.” One of her favorite examples illustrates how students can connect with those they would never have the opportunity to learn from in real life: “we had a medical student who tweeted a question about how the hormone aldosterone worked, and there was a flood of responses to this first-year student from incredibly senior people in the field. Being able to connect with these role models in the field would not be possible in the real world.” For the journal club, they also invite the journal authors to participate, which they often do. This aspect adds a unique perspective to the discussion as participants are able to ask questions and gain insight from the author themselves. This opportunity would be scarce in a face-to-face setting.

Furthermore, Farouk has harnessed opportunities to foster mentorship for trainees interested in nephrology by connecting students with volunteer faculty mentors in a virtual setting: “the goal of our virtual nephrology mentoring program (NephSIM Nephrons) is to provide a tailored learning experience for trainees through the provision of mentorship and networking opportunities. Trainees worldwide are assigned into small groups and matched with 2 - 3 mentors who are leaders in their field. The groups meet regularly throughout the year, fostering close connections and engaging in ongoing discussions to build their knowledge, skills, and networks. Group meetings would not have been possible otherwise as experts are spread across the world and travel would have made this type of approach difficult if not impossible for most students.” While Farouk helps trainees to foster connections both at scale in the Twitterverse and small mentored groups, they are also helping to support each other and developing their own creative ways to connect.

### Case Study: Keeping in Touch as Real People

#### Rebecca O’Rourke, Postgraduate Research Studies Lead, Institute of Medical Education [LIME], School of Medicine, University of Leeds

During the COVID-19 lockdown, evidence suggested that social isolation and anxiety among students increased [43, 44]. Rebecca O’Rourke was concerned for her postgraduate research students (PGRs) that transferring teaching and development activities, such as workshops, journal clubs, and supervision meetings, to an online environment might increase these feelings of social isolation. Attempting to address this, she offered a weekly informal Keep in Touch [KiT] meeting: “KiT meetings were non-compulsory although a core group attended most meetings and others came intermittently. These were informal meetings, facilitated through open questions and anecdotes and there was no expectation of preparing for the meeting. People just turned up to talk about their personal lives rather than their lives as researchers, although sometimes the two overlapped. We talked and listened - about books, films, progress with running challenges; about how the pandemic was impacting in the different countries and cities that we were based in; about what we were eating and cooking and about our cultures.”

KiT also provided space to acknowledge the difficulties many people had to deal with during the pandemic: illness and death, worries about parenting or family, and depression. “KiT also became a space for conversations that might otherwise have not happened – much more personal conversations about our families and cultures which, because they were happening in and across a group of between 6 and 10 people, drew out things we had in common and areas of difference. Listening was as important as talking; although people were invited to talk they were not forced to do so.” This highlighted another new role for faculty in being able to identify PGRs who needed additional support services and having these contacts on hand to be able to refer students who needed this additional support.

Importantly, there was a crossover from the informal interactions in KiT that benefited the more formal PGR activities “there was a frankness in discussion and challenge in the formal meetings that seemed to be encouraged by the trust emerging from apparently inconsequential conversations.” For O’Rourke, KiT exchanges specifically highlighted how little she understood the lives of the PGRs: “I realized, watching their academic confidence develop, how important it was to cultivate authentic and holistic engagement with them as people in order to create space for their research to flourish. This worked both ways, with PGRs commenting that as all the online activities took us into each other’s homes the roles and identity of faculty/mentor/student became more fluid. This was described by one PGR as ‘seeing the person as a real person’.”

Ironically, the online space removed the busyness and competing time demands and schedules of the shared physical environment that kept learners at a distance. So, both social and academic activities were enriched by the inclusion of part-time PGRs for whom employment, the return to clinical activities or geographical distance prevented attendance on campus. PGRs also took the initiative themselves to broaden their supportive community of practice [45], facilitating social connection through organized online book clubs and film nights that facilitated new connections and friendships.

O’Rourke did not plan to provide peer or faculty-led student support online; instead, it developed organically. “If we have advice for anyone wishing to follow our example it would be to trust each other and the process – genuine informality and spontaneity has been really important. PGRs often attend with babies in arms, while getting ready for work, or when sitting in their garden. However, the process needs some shaping and we recommend agreeing to some ground rules around practicalities, like the option to have the camera on or off and respecting those who prefer to listen rather than talk. Our group, which included 2 PGRs who started their studies during COVID-19 and have only met group members online, intuitively found the give and take of non-judgmental conversation. Another group might need to discuss turn-taking, appropriate and inappropriate comments. Recording also needs to be discussed. We record the academic online sessions but not KiT. The most important advice we can offer is to maintain a supportive and friendly atmosphere.”

Both Farouk and O’Rourke’s experiences highlighted the extent to which social interaction and engagement underpin and integrate academic development and show the potential an online environment has to create community and connection. They also challenge our assumptions about the limits and possibilities of both face-to-face and online environments.

## Conclusion

As online educators continue to integrate evidence, experience, and reflection rooted in appreciative inquiry, we provide case examples of practical, impactful, and strength-based ways to undo the myths surrounding online learning. We have seen faculty growth in terms of learning about and applying evidence-based approaches to online course design and teaching, balancing student well-being with academic rigor by striving toward a more compassionate approach, and building community and connection at a distance. While the educational approaches presented here are nonexhaustive, we hope that the real-life examples inspire colleagues to share their own effective practices to enhance online teaching and learning experience.

## References

[1] Sandars J, Correia R, Dankbaar M, et al. Twelve tips for rapidly migrating to online learning during the COVID-19 pandemic. MedEdPublish 2020; 9(1). 10.15694/mep.2020.000082.1 PMC10697562 38058947

[2] Grant M, Thornton H. Best Practices in Undergraduate Adult-Centered Online Learning: Mechanisms for Course Design and Delivery. MERLOT Journal of Online Learning and Teaching 2007; 3: 346–356.

[3] Cutri RM, Mena J. A critical reconceptualization of faculty readiness for online teaching. Distance Education 2020; 41(3): 361–380.

[4] Picciano A. Theories and Frameworks for Online Education: Seeking an Integrated Model. Online Learning 2017; 21(3).

[5] Mayes T, De Freitas S. Review of e-learning theories, frameworks and models. London: Joint Information Systems Committee, 2004.

[6] Bates AW. *Managing Technological Change: Strategies for College and University Leaders*. San Francisco: Jossey-Bass, 2000.

[7] Clark RE. Learning from media. Greenwich, CT: Information Age Publishing, 2001.

[8] Quinn CN. Millennials, goldfish & other training misconceptions: Debunking learning myths and superstitions. Alexandria, VA: ATD Press, 2018.

[9] Bushe GR. Foundations of Appreciative Inquiry: History, Criticism and Potential. AI Practitioner 2012; 14(1): 8–20.

[10] Denhardt RB, Denhardt JV, Aristigueta MP, Rawlings KC. Managing human behavior in public and nonprofit organizations. Washington, DC: CQ Press, 2020.

[11] Cooperrider D, Srivastva S. Appreciative Inquiry in Organizational Life. In: Woodman RW, Pasmore WA, eds. Research in Organizational Change and Development. Stamford, CT: JAI Press, 1987, pp. 81–142.

[12] Boyce ME. Organizational Learning Is Essential to Achieving and Sustaining Change in Higher Education. Innovative Higher Education 2003; 28(2): 119–136.

[13] Cooperrider DL, Whitney D. A positive revolution in change: Appreciative Inquiry. In: Holman P, Devane T, eds. Appreciative Inquiry. San Francisco, CA: Barrett-Koehler Communications, Inc, 1999.

[14] He W, Xu G, Kruck S. Online IS Education for the 21st Century. Journal of Information Systems Education 2014; 25: 101–106.

[15] Mirhosseini F, Mehrdad N, Bigdeli S, Peyravi H, Khoddam H. Exploring the concept of scholarship of teaching and learning (SoTL): Concept analysis. Medical Journal of the Islamic Republic of Iran 2018; 32: 96. 31024863 10.14196/mjiri.32.96 PMC6477884

[16] Bierne E, Romanoski MP. Instructional Design in Higher Education: Defining an Evolving Field. OLC Outlook: An Environmental Scan of the Digital Learning Landscape, 2018.

[17] Clark RCM, Mayer RE. e-Learning and the Science of Instruction(4th edition). Hoboken, NJ: Wiley, 2016.

[18] Mayer RE. Multimedia Learning(3rd edition). New York: Cambridge University Press; 2020.

[19] Mayer RE, Fiorella L., eds. The Cambridge Handbook of Multimedia Learning(3rd edition). New York: Cambridge University Press, in press.

[20] Sweller J. CHAPTER TWO - Cognitive Load Theory. In: Mestre JP, Ross BH, eds. Psychology of Learning and Motivation. Vol 55: Academic Press, 2011, pp. 37–76.

[21] Lee J. A neuropsychological exploration of zoom fatigue. *Psychiatric Times,* November 17, 2000 [Internet] [cited April 13, 2021]. (https://www.psychiatrictimes.com/view/psychological-exploration-zoom-fatigue)

[22] King A. From Sage on the Stage to Guide on the Side. College Teaching 1993; 41(1): 30–35.

[23] Kapp F, Proske A, Narciss S, Körndle H. Distributing vs. Blocking Learning Questions in a Web-Based Learning Environment. Journal of Educational Computing Research 2015; 51(4): 397–416.

[24] Wadsworth BJ. Piaget’s theory of cognitive and affective development: foundations of constructivism. White Plains, NY: Longman Publishers, 1996.

[25] Burns D, Dagnall N, Holt M. Assessing the Impact of the COVID-19 Pandemic on Student Wellbeing at Universities in the United Kingdom: A Conceptual Analysis. Frontiers in Education 2020; 5(204).

[26] Burki TK. COVID-19: consequences for higher education. Lancet Oncology 2020; 21(6): 758–758. 32446322 10.1016/S1470-2045(20)30287-4 PMC7241990

[27] Baran E, AlZoubi D. Human-centered design as a frame for transition to remote teaching during the COVID-19 pandemic. Journal of Technology and Teacher Education 2020; 28(2): 365–372.

[28] CAST. Universal Design for Learning Guidelines, version 2.2. 2018.

[29] Davidson S. Trauma-informed Practices for Post-Secondary Education: A Guide. Portland, OR: Education Northwest, 2017.

[30] Carello J, Butler LD. Potentially Perilous Pedagogies: Teaching Trauma Is Not the Same as Trauma-Informed Teaching. Journal of Trauma & Dissociation 2014; 15(2): 153–168. 24313321 10.1080/15299732.2014.867571

[31] Hsu H-CK, Wang CV, Levesque-Bristol C. Reexamining the impact of self-determination theory on learning outcomes in the online learning environment. Education and Information Technologies 2019; 24(3): 2159–2174.

[32] Anderson T, Rourke L, Garrison D, Archer W. Assessing Teaching Presence in a Computer Conferencing Context. Journal of Asynchronous Learning Networks 2001; 5.

[33] Brinkerhoff J, Koroghlanian CM. Online Students’ Expectations: Enhancing the Fit between Online Students and Course Design. Journal of Educational Computing Research 2007; 36(4): 383–393.

[34] Paechter M, Maier B, Macher D. Students’ expectations of, and experiences in e-learning: Their relation to learning achievements and course satisfaction. Computers & Education 2010; 54(1): 222–229.

[35] Shaw M, Clowes M, Burrus S. A Comparative Typology of Student and Institutional Expectations of Online Faculty. Ubiquitous Learning: An International Journal 2017; 10: 1–9.

[36] Bransford D, Brown A, Cocking R. How People Learn: Brain, Mind, Experience and School. Washington, DC: National Academies Press, 1999.

[37] Ni AY. Comparing the Effectiveness of Classroom and Online Learning: Teaching Research Methods. Journal of Public Affairs Education 2013; 19(2): 199–215.

[38] Irani TA, Wilson SB, Slough DL, Rieger M. Graduate student experiences on- and off-campus: Social connectedness and perceived isolation. International Journal of E-Learning and Distance Education 2014; 28(1).

[39] Shea P, Li CS, Swan K, Pickett A. Developing learning community in online asynchronous college courses: The role of teaching presence. Online Learning 2019; 9(4).

[40] Haythornthwaite C, Kazmer MM, Robins J, Shoemaker S. Community Development Among Distance Learners: Temporal and Technological Dimensions. Journal of Computer-Mediated Communication 2000; 6(1).

[41] Dave NN, Sparks MA, Farouk SS. An introduction and guide to becoming a social media savvy nephrologist. *Nephrology Dialysis Transplantation* 2020; gfaa067.

[42] Rein JL, Sparks MA, Hilburg R, Farouk SS. Tackling acid-base disorders, one Twitter poll at a time. Advances in Physiology Education 2020; 44(4): 706–708. 33079564 10.1152/advan.00099.2020

[43] Chirikov I, Soria KM, Horgos B, Jones-White D. Undergraduate and Graduate Students’ Mental Health During the COVID-19 Pandemic. UC Berkeley: Center for Studies in Higher Education, 2020.

[44] Woolston C. Signs of depression and anxiety soar among US graduate students during pandemic. Nature 2020; 585: 147–148. 32811983 10.1038/d41586-020-02439-6

[45] Wenger E, McDermott RA, Snyder W. Cultivating Communties of Practice: A Guide to Managing Knowledge. Boston, MA: Harvard Business School Press, 2002.

